# Evaluation of Antibiotic Resistance Mechanisms in Gram-Positive Bacteria

**DOI:** 10.3390/antibiotics13121197

**Published:** 2024-12-08

**Authors:** Pratiksing Rajput, Kazi S. Nahar, Khondaker Miraz Rahman

**Affiliations:** 1 Institute of Pharmaceutical Science, King’s College London, 150 Stamford Street, London SE1 9NH, UK; pratiksingh67489@gmail.com; 2 Department of Natural Sciences, Faculty of Science & Technology, Middlesex University, The Burroughs, Hendon, London NW4 4BT, UK; k.nahar@mdx.ac.uk

**Keywords:** antimicrobial resistance (AMR), priority pathogen, gram-positive, antibiotics, multidrug resistance (MDR), *S. aureus*, *S. pneumoniae*, *E. faecium*

## Abstract

The prevalence of resistance in Gram-positive bacterial infections is rapidly rising, presenting a pressing global challenge for both healthcare systems and economies. The WHO categorizes these bacteria into critical, high, and medium priority groups based on the urgency for developing new antibiotics. While the first priority pathogen list was issued in 2017, the 2024 list remains largely unchanged. Despite six years having passed, the progress that has been made in developing novel treatment approaches remains insufficient, allowing antimicrobial resistance to persist and worsen on a global scale. Various strategies have been implemented to address this growing threat by targeting specific resistance mechanisms. This review evaluates antimicrobial resistance (AMR) in Gram-positive bacteria, highlighting its critical impact on global health due to the rise of multidrug-resistant pathogens. It focuses on the unique cell wall structure of Gram-positive bacteria, which influences their identification and susceptibility to antibiotics. The review explores the mechanisms of AMR, including enzymatic inactivation, modification of drug targets, limiting drug uptake, and increased drug efflux. It also examines the resistance strategies employed by high-priority Gram-positive pathogens such as *Staphylococcus aureus*, *Streptococcus pneumoniae*, and *Enterococcus faecium*, as identified in the WHO’s 2024 priority list.

## 1. Antimicrobial Resistance (AMR)

Antimicrobial resistance (AMR) poses a significant global health threat as it involves the adaptation of microorganisms like bacteria, viruses, fungi, and parasites to withstand the effects of drugs. This resistance renders conventional treatments ineffective, thereby escalating the threat to human health. Antimicrobial agents include antibiotics, antivirals, antifungals, antibacterial, and antiparasitic drugs. These drugs are crucial in the fight against infectious diseases. The application of antimicrobial agents to treat and prevent infections has driven an evolutionary response in microorganisms, leading to the emergence of drug resistance [1]. This occurs when bacterial pathogens become insensitive to high doses of multiple antibiotic classes or become unresponsive to lethal doses of antibiotics. This form of resistance, known as multidrug resistance (MDR), poses a significant challenge to the effectiveness of antibiotics in treating infectious diseases [2].

Antimicrobial resistance has intensified the global health crisis by increasing rates of illness and death, primarily due to bacterial strains developing resistance to antibiotics [3]. This global threat has been driven by the excessive and improper use of antimicrobial substances in both humans and animals. Among the contributing factors are the excessive administration of antibiotics in situations where they are not clinically necessary [4], the distribution of low-quality antibiotics [5], and the prescription of antibiotics for viral infections like colds or flu by physicians and pharmacists [6]. In many developing countries, antibiotics are readily available over the counter, enabling self-treatment even when unwarranted [7]. Nosocomial infections or hospital-acquired infections are also significant contributors to antimicrobial resistance. In hospital settings, antibiotics are employed not solely for therapeutic purposes but also as a preventive measure during surgeries and medical procedures to reduce infection risk [5]. The increase in hospital-acquired infections is attributed to cross-contamination between patients, inadequate hygiene practices, insufficient use of gloves, and the unregulated overuse of antibiotics [8]. The overuse of antibiotics in livestock further exacerbates AMR. Antibiotics are administered to animals not only to treat illnesses and prevent infections but also for non-therapeutic purposes, such as promoting growth. The close relationship between humans and animals increases the risk of transmitting antibiotic-resistant bacteria from animals to humans [9]. Additionally, the overuse and misuse of antibiotics contribute to environmental contamination. When antibiotics are ingested, enzymes in the body break them down into active compounds that halt microbial growth and treat infections. However, residual antibiotics are excreted through urine and feces, entering the environment via sewage systems and raising antibiotic concentrations in natural ecosystems. This excessive presence of antibiotics in the environment drives bacterial evolution through a selective process, enabling pathogens to mutate and develop resistance to available antibiotics [10,11].

The rapid increase in antimicrobial resistance (AMR) worldwide is driven by the overuse of antimicrobial agents. This growing crisis is significantly affecting the human population, resulting in prolonged treatment durations and impacting a larger number of individuals. Consequently, older and slower medical techniques, such as isolation, debridement, disinfection, and even amputation, may need to be employed due to the diminishing effectiveness of antimicrobial treatments [1]. Individuals suffering from infections due to antibiotic-resistant bacteria need extended hospital stays and more intensive treatments, leading to prolonged recovery times and a heightened risk of complications [12]. When first-line therapies prove ineffective, patients are often administered second- or third-line treatments, which are typically more hazardous and costly [13]. The use of these additional drugs, combined with the need for more diagnostic tests and prolonged hospital care, significantly increases healthcare expenses.

Statistics show that antimicrobial resistance (AMR) and multidrug resistance (MDR) are escalating global health concerns with significant implications for public health. Each year, bacterial infections result in approximately 7.7 million deaths, of which 4.95 million are associated with drug-resistant pathogens, and 1.27 million are directly attributed to bacteria resistant to available antibiotics [7]. In Europe, the financial burden of antibiotic resistance is substantial, estimated at a minimum of EUR 1.5 billion, with over EUR 900 million attributed to hospital-related costs. In the United States, the Centers for Disease Control and Prevention (CDC) estimates the annual economic impact of AMR to be approximately USD 55 billion. This includes USD 20 billion in direct healthcare costs and an additional USD 35 billion in societal costs due to lost productivity. These figures highlight the significant financial strain AMR places on healthcare systems and society at large, with hospital expenses in Europe and direct healthcare costs in the U.S. being major contributors. Furthermore, the reduction in productivity caused by AMR-related health issues exacerbates this economic challenge [13].

**Figure 1:**
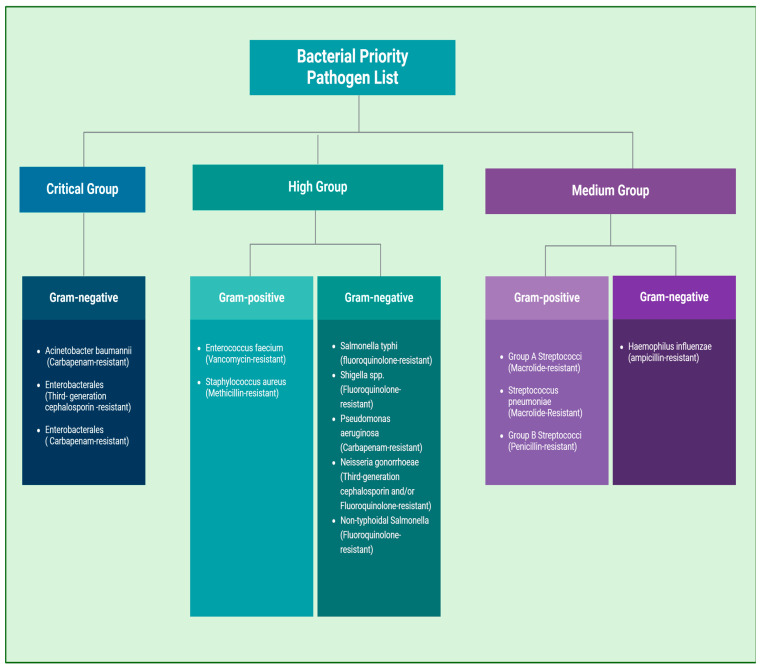
WHO Bacterial Priority Pathogen List (BPPL), 2024.

The World Health Organization (WHO) recently released a global priority list of pathogens, categorizing them into three groups based on the urgency for new antibiotics: critical, high, and medium priority (Figure 1). This classification was determined through a systematic evaluation process using a comprehensive multi-criteria decision analysis (MCDA) method. The approach involved input from an expert panel, which assessed each pathogen-antibiotic pairing based on eight critical factors: mortality, incidence, non-fatal health burden, resistance trends, transmissibility, preventability in healthcare settings and the community, treatability, and the antibacterial pipeline [14]. These categories highlight antibiotic-resistant bacteria that urgently require new research and treatment development to address the growing threat of antimicrobial resistance. The initiative aims to focus scientific efforts and resources on developing effective treatments for these dangerous pathogens, thereby reducing the global impact of resistant infections. Among the pathogens listed on the WHO’s 2024 priority list, notable Gram-positive bacteria include Methicillin-resistant *Staphylococcus aureus* (MRSA) and Vancomycin-resistant *Enterococcus faecium* (VRE), both categorized as high priority. MRSA is a significant concern due to its resistance to multiple antibiotics, while VRE poses a serious threat because of its resistance to vancomycin and other antimicrobial agents. Additionally, Group A *Streptococcus* (GAS), Group B *Streptococcus* (GBS), and *Streptococcus pneumoniae* are identified as medium-priority pathogens. This classification underscores the urgent need for ongoing research and the development of new treatments to combat these resistant bacteria [14].

The list classifies bacteria into critical, high, and medium-priority groups to guide research and development (R&D) efforts and public health measures against antimicrobial resistance [14].

## 2. Antimicrobial Resistance in Gram-Positive Bacteria

Bacterial resistance can be either natural, always expressed in a species, or adaptive, expressed only in the presence of therapeutic antibiotics [15]. The origin of resistance in bacteria stems from diverse evolutionary pathways in bacterial genomes, primarily horizontal gene transfer (HGT) and genetic mutations [16]. Resistance can emerge from natural mutations in bacterial genetic material. These mutations typically affect specific categories of genes, including those responsible for drug targets, drug transporters, regulatory factors governing drug transporters, and enzymes that modify antibiotics [15,17]. Resistance resulting from such acquired mutations varies widely in complexity and mechanisms. HGT is another critical driver of bacterial evolution. Many antimicrobial agents used in medical settings originate from natural sources, primarily environmental, and ultimately return to the environment, particularly soil. Environmental bacteria exposed to these antimicrobial agents serve as a source of resistance-causing genes [18]. This reservoir of resistance genes, known as the “environmental resistome”, provides clinically relevant bacteria with a significant source from which to acquire antibiotic resistance genes [19]. Additionally, bacteria can acquire resistance genes from other bacterial species through mechanisms such as conjugation, transformation, and transduction [20]. This genetic exchange accelerates the spread of resistance traits among bacterial populations, contributing to the widespread emergence of antibiotic resistance.

Resistance in Gram-positive bacteria presents a significant challenge in healthcare, often adversely affecting treatment outcomes. To address this issue, it is essential to explore the diverse mechanisms these bacteria use to resist antimicrobial agents. Understanding these mechanisms is crucial for developing novel therapeutic strategies to overcome resistance and improve patient care.

**Figure 2:**
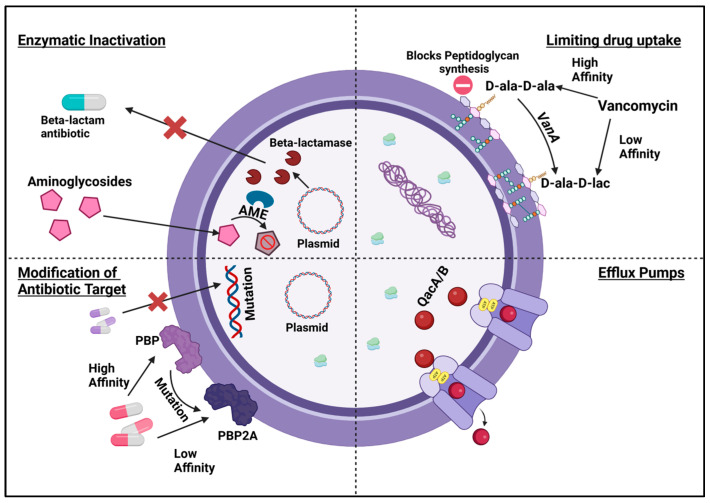
Resistance mechanisms of Gram-positive bacteria. Figure 2 depicts four major resistance mechanisms discussed in the review, including beta-lactamase action, AMEs inactivating aminoglycosides, a mutation in the ribosomal binding site, PBP alteration, efflux pump, and cell wall modification. (Figure created using Biorender, https://www.biorender.com/).

Antibiotic resistance mechanisms can be characterized into four main groups (Figure 2):

1. Enzymatic inactivation;
2. Modification of drug target;
3. Limiting drug uptake;
4. Increased Drug efflux.

Gram-positive bacteria employ various defense mechanisms, including alterations to their cell wall structure and the production of enzymes that deactivate antibiotics. Additionally, they can develop resistance through the use of efflux pumps that expel antibiotics, modifications to penicillin-binding proteins (PBPs), and the formation of biofilms. Understanding these mechanisms is crucial for healthcare professionals and researchers, as it enables the development of targeted interventions to combat antimicrobial resistance effectively.

### 2.1. Enzymatic Inactivation or Modification

The inactivation or modification of drugs by Gram-positive bacteria is an irreversible resistance mechanism that occurs primarily through two processes: enzymatic degradation of the drug or the transfer of a chemical group to the antibiotic. Enzymatic degradation is primarily carried out by β-lactamases, a large group of drug-hydrolyzing enzymes that degrade antibiotics by hydrolyzing the β-lactam ring [21]. This mechanism is commonly used against β-lactam and tetracycline antibiotics. Drug modification through the transfer of chemical groups—such as acetyl, phosphoryl, or adenyl groups—is another resistance strategy employed against aminoglycosides, chloramphenicol, streptogramins, and fluoroquinolones. This process, facilitated by transferases, involves phosphorylation, acetylation, or adenylation of the antibiotic [15]. Aminoglycoside-modifying enzymes, which include various transferases, confer high levels of resistance by obstructing the antibiotics’ ability to bind to their target sites [22]. Antibiotics exert their effects by binding strongly to specific bacterial targets, enabling them to enter the bacteria and perform their antimicrobial functions. However, when these bacterial targets undergo modifications, the antibiotics’ ability to bind effectively is compromised, leading to a diminished capacity to inhibit bacterial growth and activity [23].

#### 2.1.1. Direct Inactivation by Beta-Lactamases

Beta-lactamases are enzymes synthesized by certain bacteria to confer resistance to β-lactam antibiotics. These antibiotics function by disrupting bacterial cell wall formation, binding covalently to penicillin-binding proteins (PBPs), which are essential for the final stages of peptidoglycan cross-linking. The primary mechanism of resistance to β-lactam antibiotics involves the bacterial production of β-lactamase enzymes, which break down the peptide bond in the four-membered β-lactam ring, thereby neutralizing the antibiotic’s effectiveness [24]. To date, over 300 beta-lactamase enzymes have been identified and classified into four classes (A, B, C, and D) based on sequence similarity and catalytic mechanisms. The first crystal structure determined was for a class A beta-lactamase from *Staphylococcus aureus* PC1. Class B, or metallo-beta-lactamases, are further divided into subgroups B1, B2, and B3 based on their metal ion requirements. Class C beta-lactamases are exclusively produced by Gram-negative bacteria. Class D includes OXA enzymes, which lack effective inhibitors, posing a significant challenge for clinical resistance management [25]. To mitigate the impact of beta-lactamases, extended-spectrum beta-lactam antibiotics, such as ceftazidime and cefotaxime, have been developed, along with beta-lactamase inhibitors like sulbactam and clavulanic acid [24].

#### 2.1.2. Modification of Drug by Chemical Group Transfer

Antibiotic resistance can occur when bacterial enzymes add chemical groups to antibiotic molecules at susceptible sites, creating steric hindrance that prevents the antibiotic from binding to its target protein. This mechanism is mediated by the production of Aminoglycoside-Modifying Enzymes (AMEs). There are three main classes of AMEs: acetyltransferases, phosphotransferases, and nucleotidyltransferases. *N*-acetyltransferases (AACs) acetylate an amino group using acetyl-Coenzyme A, *O*-nucleotidyltransferases (ANTs) transfer an adenyl group from ATP to a hydroxyl group on the antibiotic, and O-phosphotransferases (APHs) phosphorylate a hydroxyl group, also using ATP [26]. A study demonstrated high levels of agreement between hybridization results and the enzyme content deduced for various aminoglycoside-modifying enzymes. The results revealed a strong correlation between the presence of these enzymes and resistance phenotypes. For instance, ANT(6) (aminoglycoside nucleotidyltransferase) showed 80% and 87.6% agreement in *Staphylococcus* and *Enterococcus* species, respectively, indicating its significant role in resistance. Moreover, ANT(4′) demonstrated perfect agreement (100% for both species), underscoring its universal presence in clinical isolates and its substantial contribution to resistance. These findings emphasize the urgency of developing inhibitors or alternative drugs capable of bypassing or neutralizing these enzymes to address antibiotic resistance effectively [27].

### 2.2. Modification of Drug Target

Gram-positive bacteria resist antibiotics primarily by altering their drug targets. For example, methicillin-resistant *Staphylococcus aureus* (MRSA) modifies its penicillin-binding proteins (PBPs), preventing beta-lactam antibiotics from binding effectively [19]. Other modifications include alterations to ribosomal RNA (rRNA) or ribosomal proteins, which affect the binding sites for antibiotics such as macrolides, lincosamides, and streptogramins [28].

#### 2.2.1. PBP Alteration

β-Lactam antibiotics target enzymes involved in cell wall synthesis, known as penicillin-sensitive enzymes. These enzymes are identified by their covalent binding to radio-labeled penicillin, which is why they are called penicillin-binding proteins (PBPs) [29]. PBPs are located in the bacterial cell membrane and play a crucial role in the final stages of murein (peptidoglycan) synthesis, as mentioned above. These proteins are essential for constructing and maintaining the bacterial cell wall. β-Lactam antibiotics, such as penicillin, inhibit PBPs by mimicking the structure of the natural pentapeptide substrate. By competing for and binding to the active site of these enzymes, β-lactam antibiotics prevent PBPs from performing their role in cell wall synthesis, resulting in the weakening and eventual lysis of the bacterial cell [30]. Modified PBPs linked to β-lactam resistance are more frequently observed in Gram-positive bacteria compared to Gram-negative bacteria [29]. β-Lactam antibiotics act by acylating PBPs and inactivating them, thereby preventing the transpeptidation of peptidoglycan. This disruption ultimately weakens the bacterial cell wall. Penicillin achieves this by functioning as a structural analog and forming an irreversible penicilloyl–enzyme complex, similar to the transient acyl-enzyme intermediate generated during normal transpeptidation. Despite the long-standing effectiveness of β-lactam antibiotics, the emergence of drug-resistant bacterial strains has become a significant global issue [30].

#### 2.2.2. Modification of Ribosomal Binding Sites

Modifications to ribosomal target sites are a critical mechanism by which Gram-positive bacteria develop antibiotic resistance. This resistance typically involves mutations in the genes encoding ribosomal RNA (rRNA) or ribosomal proteins, which alter the binding sites for antibiotics on the ribosome. Such ribosomal mutations have been identified in several clinically relevant Gram-positive pathogens, including *Staphylococcus aureus* and *Streptococcus pneumoniae* [31].

### 2.3. Limiting Drug Uptake

Limiting drug uptake is a key resistance mechanism employed by Gram-positive bacteria. They exhibit resistance to antimicrobial agents through several strategies, particularly by reducing drug uptake. These strategies include modifications to the cell wall or membrane permeability and the formation of biofilms [21].

#### 2.3.1. Modification of Cell Wall or Membrane Permeability

Over time, Gram-positive bacteria have developed various adaptations to modify their membrane and cell wall structures. These changes are critical for their survival when exposed to antibiotics, making bacterial infections increasingly difficult to treat effectively. These adaptations enhance resistance to antimicrobial agents and promote the spread of resistant strains [32]. Unlike Gram-negative bacteria, Gram-positive bacteria are less likely to employ mechanisms that limit drug uptake because they lack an outer membrane composed of lipopolysaccharides (LPSs), which serve as a common barrier in Gram-negative bacteria. Instead, Gram-positive bacteria possess a thick peptidoglycan layer that does not provide the same restrictive barrier to antibiotic entry as the outer membrane in Gram-negative bacteria [15,22].

#### 2.3.2. Biofilm Formation

**Figure 3:**
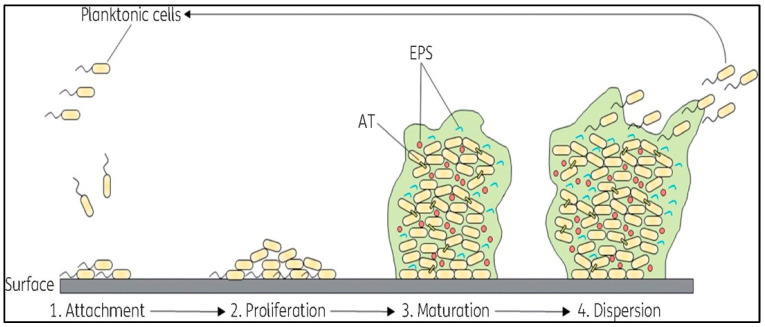
Stages of biofilm formation and development. This illustration depicts the sequential stages of biofilm formation on a surface by planktonic bacterial cells. Adapted from [37].

A biofilm is a community of bacteria enclosed in a polymer matrix composed of polysaccharides, proteins, and DNA, which are produced by the bacteria themselves. These biofilms contribute to persistent infections due to their heightened resistance to antibiotics [33]. Biofilm formation occurs through a series of steps, including conditioning, attachment, growth, metabolism, and, finally, dispersion, which enables bacteria to colonize and develop (Figure 3) [34]. Biofilms are associated with various human infections, such as urinary tract infections, endocarditis, chronic ear infections, gastrointestinal ulcers, and osteomyelitis. Within biofilm communities, bacteria communicate by releasing and detecting chemical signals in a process known as quorum sensing (QS), which is regulated by population density [35]. QS controls the synthesis of virulence factors crucial to the pathophysiology of infections, including cellular lysins (such as rhamnolipid) and extracellular enzymes [33]. Approximately 80% of human infections originate from biofilms, which demonstrate remarkable resilience against environmental factors, antimicrobial agents, disinfectants, and the body’s immune responses [36]. Eliminating fully developed bacterial biofilms is extremely challenging, necessitating the exploration of additional strategies and the development of innovative compounds [34].

### 2.4. Increased Drug Efflux

Certain bacteria evade antibiotics by preventing their entry into the cell and actively expelling them. This is achieved using efflux pumps, which are found in all living organisms. In bacteria, the genes encoding these efflux pumps can be located on either the chromosome or plasmids. These transport proteins work by removing harmful substances, including antibiotics, from within the bacterial cell. This action reduces the intracellular concentration of the drugs, enabling the bacteria to survive. Bacterial efflux systems are generally classified into five families: the major facilitator superfamily (MFS), the ATP-binding cassette (ABC) family, the resistance-nodulation-division (RND) family, the small multidrug resistance (SMR) family, and the multidrug and toxic compound extrusion (MATE) family [38,39,40]. Gram-positive bacteria possess four types of efflux pumps: MFS, ABC, SMR, and MATE. In contrast, the RND (resistance-nodulation-division) family of efflux pumps is unique to Gram-negative bacteria [41].

#### 2.4.1. Major Facilitator Superfamily (MFS)

Efflux pumps of the major facilitator superfamily (MFS) play a critical role in providing antibiotic resistance in Gram-positive bacteria. The MFS operates through various transport modes, including symport, antiport, and uniport [42]. These proteins typically consist of 400 to 600 amino acids, forming 12 or 14 transmembrane helices [43]. Among the MFS efflux pumps, the most extensively studied are NorA from Staphylococcus aureus and PmrA from Streptococcus pneumoniae [44].

#### 2.4.2. ATP-Binding Cassette (ABC) Family

**Figure 4:**
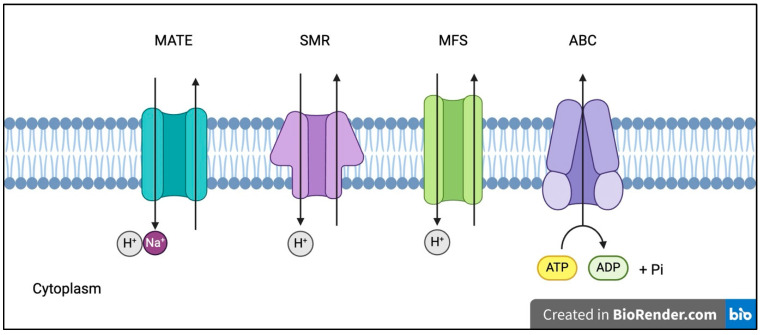
Efflux pumps in Gram-positive bacteria. Figure 4 depicts four different types of efflux pumps: multidrug and toxin extrusion (MATE) family, small multidrug resistance (SMR) family, major facilitator superfamily (MFS), and ATP-binding cassette (ABC) family.

ATP-binding cassette (ABC) systems are found in all living organisms and serve various roles in bacterial functions. In eukaryotes, ABC transporters are notable for their involvement in genetic disorders and multidrug resistance. These transporters are composed of two primary components: two transmembrane domains (TMDs), which form the channel for substance transport, and two nucleotide-binding domains (NBDs), which face the cytoplasm and play a crucial role in ATP hydrolysis [45] (Figure 4). The energy derived from ATP hydrolysis in the NBDs drives conformational changes in the TMDs, creating a high-affinity site for drugs on the inner surface of the membrane. This allows the pump to transport substrates and other materials from inside the cell to the exterior [46]. Conversely, when ADP is bound, a low-affinity site for substrates forms on the outer surface of the protein. The hydrolysis of ATP facilitates the movement of the drug from the inner leaflet of the membrane to the exterior, where it is released. Once the process is complete, ATP can bind to the other NBD, restarting the cycle [47]. This mechanism is essential for the efflux of antibiotics, toxins, and other harmful compounds, playing a vital role in bacterial resistance.

#### 2.4.3. Small Multidrug Resistance (SMR) Family

The small multidrug resistance (SMR) family includes small, homologous proteins that are 104 to 115 amino acids in length and contain four transmembrane segments [48,49]. These proteins function either as homodimers or homotetramers, with each subunit contributing to the pathway used for expelling substrates. SMR efflux pumps are transport proteins that use the proton motive force across the cell membrane to remove various toxic substances from the cell. Their typical mechanism involves an antiport system, in which protons (H+) entering the cell are exchanged for drugs being expelled outward [50].

#### 2.4.4. Multidrug and Toxin Extrusion (MATE) Family

Multidrug and toxin extrusion (MATE) efflux pumps in Gram-positive bacteria function as multidrug resistance (MDR) transporters. These pumps play a critical role in removing harmful substances, such as antibiotics, from bacterial cells, contributing to the development of antibiotic resistance. Structurally, MATE pumps consist of twelve transmembrane helices arranged in two sets of six, connected by a cytoplasmic loop [51]. Unlike many other efflux pump families that rely on proton gradients, MATE pumps utilize a sodium ion gradient, functioning as sodium/drug antiporters [52]. Sodium ions drive the expulsion of multiple drugs by inducing conformational changes in the protein rather than directly competing with the amino acids at the substrate-binding site [53] (Figure 4).

## 3. Mechanism of Resistance in WHO Priority Gram-Positive Pathogens

The review will focus on antibiotic resistance mechanisms in WHO priority pathogens, mainly Gram-positive bacteria involving *Staphylococcus aureus*, *Streptococcus pneumonia,* and *Enterococcus faecium.*

### 3.1. Staphylococcus aureus

*S. aureus* is a Gram-positive species that belongs to the family Micrococcaceae. It is commonly found on human skin and in the nose. This bacterium can cause a wide range of infections affecting various parts of the body, including the skin, soft tissues, and internal organs. These infections can be severe and may lead to significant illness or even death. *S. aureus* produces various proteins both on its cell surface and outside the cell, contributing to its ability to cause disease. It remains a significant concern in both communities and hospitals due to its capacity to cause widespread infections. In hospitals, patients are particularly vulnerable to *S. aureus* infections, especially in surgical wounds and medical devices. The bacteria can colonize these devices, leading to localized damage or the spread of infection throughout the body. Additionally, consuming food contaminated with toxins produced by *S. aureus* can result in food poisoning, underscoring the importance of preventing its spread [54].

Over the past 20 years, there has been a notable increase in staphylococcal infections in both community and hospital settings [55]. Methicillin-resistant *Staphylococcus aureus* (MRSA) has been on the World Health Organization’s (WHO) high-priority pathogen list since at least 2017. A 2014 study revealed that MRSA is widespread in many Asian hospitals, with certain countries in the region reporting some of the highest global MRSA rates. Moreover, after 2000, community-associated MRSA (CA-MRSA) emerged in most Asian countries, with some regions experiencing exceptionally high incidence rates exceeding 50% [56]. Medications commonly prescribed for *S. aureus* infections include oxacillin, nafcillin, cefazolin, and cephalothin, particularly for strains resistant to β-lactam antibiotics due to β-lactamase production. However, *S. aureus* often exhibits methicillin resistance. For MRSA infections, vancomycin is an effective treatment. When vancomycin is unsuitable, alternative options include fluoroquinolones, clindamycin, and minocycline [55].

The following section will explain the resistance mechanisms employed by *S. aureus.*

#### 3.1.1. β-Lactamases

One common mechanism of resistance in *S. aureus* involves the blaZ gene, which encodes the beta-lactamase enzyme. This enzyme hydrolyzes the beta-lactam ring, thereby deactivating β-lactamase-sensitive antibiotics [57]. In clinical strains, resistance is primarily regulated by BlaR1. This receptor detects β-lactams by acylating its sensor domain, initiating transmembrane signaling that activates the metalloprotease domain within the cell. This activation induces the expression of the blaZ gene, leading to the production of beta-lactamase [58]. A study investigating β-lactamase production and its association with antimicrobial susceptibility revealed that resistance rates ranged from 30% to 70% for various antibiotics, including tetracycline, streptomycin, augmentin, erythromycin, and gentamicin. The highest resistance rates were observed for three β-lactam antibiotics: cloxacillin (83.1%), ceftriaxone (75.7%), and amoxicillin (72.9%). Although amoxicillin/clavulanic acid and ceftriaxone are designed to counteract β-lactamase activity and enhance the efficacy of β-lactam antibiotics, the study found that *S. aureus* resistance to these drugs was significantly associated with β-lactamase production [59].

#### 3.1.2. Aminoglycoside Modification

*S. aureus* inactivates aminoglycoside antibiotics through aminoglycoside-modifying enzymes (AME), such as aminoglycoside phosphotransferase (APH), acetyltransferases (AAC), and nucleotidyltransferase (ANT) enzymes. Among *S. aureus*, the most prevalent AME-encoding genes are *aac(6′)-Ie-aph(2″), aph(3′)-IIIa, and ant(4′)-Ia*, which can be located on either plasmids or chromosomes. In *S. aureus* strains collected from burn patients, approximately 95 out of 151 isolates harbored genes encoding aminoglycoside-modifying enzymes (AME). Specifically, the *aac(6′)-Ie-aph(2″)-I* gene was found in 18 isolates, while the *aph(3′)-IIIa* and *ant(4′)-Ia* genes were detected in 8 and 6 isolates, respectively. Notably, all three genes were concurrently present in 69 isolates [60]. According to a recent study, the *aac(6′)-Ie-aph(2″)* gene was the most frequently identified gene in *S. aureus* isolates [61]. This high prevalence of AME genes highlights the widespread nature of aminoglycoside resistance and the frequent adoption of this resistance mechanism by *S. aureus*.

A study of various *S. aureus* strains revealed that the AME-encoding gene *aac(6′)-Ie/aph(2″)* uniquely confers resistance to gentamicin with a minimum inhibitory concentration (MIC) of 8 μg/mL or higher and to tobramycin with a cutoff MIC of 8 μg/mL. Strains carrying the *ant(4′)-I* gene exhibit significant resistance to tobramycin (MIC ≥ 128 μg/mL), while those harboring the *aph(3′)-III* gene display high resistance to lividomycin (MIC ≥ 1024 μg/mL) [62]. Additionally, the *aph(3′)-III* gene confers resistance to kanamycin, amikacin, and neomycin. Strains producing these enzymes are consistently resistant to kanamycin, with an MIC of 64 mg/L.

Other relevant genes include *ant(6)-Ia*, which encodes the ANT(6)-Ia nucleotidyltransferase responsible for streptomycin resistance. The *aadA5* cassette gene encodes the ANT(3″)-Ia nucleotidyltransferase, which confers resistance to both streptomycin and spectinomycin. Similarly, the *ant(9)-Ia* gene encodes the ANT(9) nucleotidyltransferase, imparting resistance to spectinomycin [63].

Arbekacin (ABK), an aminoglycoside antibiotic, is effective against MRSA; however, its use is limited due to the risk of kidney damage [64]. Studies on novel aminoglycoside derivatives, such as 2-hydroxyarbekacin, show promise in treating resistant *S. aureus* strains. This derivative has demonstrated reduced nephrotoxicity compared to ABK while exhibiting superior antibacterial properties [65].

#### 3.1.3. Modification of Cell Wall

*Staphylococcus aureus* is widely recognized for its ability to develop resistance to glycopeptide antibiotics, including vancomycin [66]. A study by Sieradzki and Tomasz demonstrated vancomycin resistance in *S. aureus* with a minimum inhibitory concentration (MIC) of 100 µg/mL [67]. Vancomycin inhibits bacterial cell wall formation by binding to the terminal d-alanine-d-alanine residues, which are essential precursors in the peptidoglycan chain of the bacterial cell membrane [68]. The *vanA* genes, located on Tn1546, encode the modification of d-alanine-d-alanine to d-alanine-d-lactate, a modified peptidoglycan precursor. This alteration significantly reduces the binding affinity of vancomycin, rendering it less effective against the modified d-alanine-d-lactate structure [69].

In clinical strains of *S. aureus* that lack van genes or changes in the terminal d-alanyl-d-alanine residues, the thickening of the cell wall is considered the primary mechanism contributing to vancomycin resistance. Increased cell wall thickness is strongly associated with the development of vancomycin resistance in vancomycin-resistant *S. aureus* (VRSA) strains. A thicker cell wall not only captures more vancomycin molecules but also reduces the time during which vancomycin can fully inhibit peptidoglycan synthesis [70]. This highlights the critical role of alterations in cell wall structures in enabling bacteria to withstand the effects of vancomycin.

Additionally, *Staphylococcus aureus* and *Enterococcus faecium* resist positively charged antimicrobials by altering their surface charge. Many cationic antimicrobial peptides (CAMPs) interact with bacterial cells through electrostatic forces on the cell surface, which are influenced by anionic elements such as phospholipids and teichoic acids in the cell membrane and wall [71]. These bacteria can neutralize the negative charge on their surface by adding a positively charged amino acid via the multipeptide resistance factor (MprF). MprF affects the sensitivity of *S. aureus* to cationic antibiotics, including the glycopeptide vancomycin, the aminoglycoside gentamicin, and the lipopeptide antibiotic daptomycin. It modifies phosphatidylglycerol, a negatively charged membrane component, by attaching L-lysine or L-alanine, thereby adding positive charges to the membrane surface and conferring resistance [72].

#### 3.1.4. Biofilm Formation

Staphylococci have long been recognized as the most common cause of biofilm-associated infections [36]. In a study on *S. aureus* isolates from hospital patients, a significant percentage of isolates (69.8% and 65.1% using the TCP and TM methods, respectively) were found to form biofilms. Among these biofilm-forming isolates, 86.7% were multidrug-resistant (MDR), whereas none of the non-biofilm producers exhibited MDR characteristics, highlighting the critical role of biofilms in promoting multidrug resistance. Furthermore, 43.3% of the biofilm producers were methicillin-resistant *S. aureus* (MRSA), compared to none of the non-biofilm producers [73].

#### 3.1.5. PBP Alteration

Certain bacteria can acquire new penicillin-binding proteins (PBPs) via horizontal gene transfer. These PBPs exhibit a reduced affinity for β-lactam antibiotics, leading to antibiotic resistance. A notable example of this mechanism is methicillin-resistant *Staphylococcus aureus* (MRSA). The *mecA* gene, which encodes PBP2A, is acquired by MRSA through horizontal gene transfer [74]. PBP2A takes over the role of peptidoglycan biosynthesis from the four native staphylococcal PBPs, as these native PBPs are highly sensitive to antibiotics and quickly undergo acylation, resulting in deactivation even at low antibiotic concentrations [75].

*S. aureus* demonstrates resistance to several antibiotics, including penicillin G, oxacillin, extended-spectrum ampicillin, piperacillin, and cephalosporins such as cefaclor, cefotaxime, cephalexin, and cefoxitin. In a study involving an *S. aureus* strain induced with IPTG, the minimum inhibitory concentrations (MICs) were significantly higher for cephalexin (64 µg/mL) and cefaclor (16 µg/mL). Moreover, there was a 16-fold increase in the MICs for both ampicillin and oxacillin in the IPTG-induced *S. aureus* strain [76].

#### 3.1.6. Modification of Ribosomal Binding Sites

In *Staphylococcus aureus*, resistance to antibiotics via modification of ribosomal binding sites involves various mechanisms. Tetracyclines inhibit protein synthesis by interfering with the ribosome’s 30S subunit. Tetracycline resistance in *S. aureus* is often mediated by Tet(M) and Tet(S) proteins, which dislodge tetracycline molecules from the 30S ribosomal subunit. Resistance to tetracyclines, such as minocycline, is specifically conferred by the Tet(M) protein [63].

A mutation in the *rplV* gene, which encodes the L22 protein in the ribosome’s 50S subunit, leads to resistance against erythromycin, quinupristin, and dalfopristin [77]. Similarly, a mutation in the *rplD* gene, which encodes the L4 protein in the same ribosomal subunit, results in resistance to erythromycin and spiramycin [78].

Changes in ribosomal proteins, particularly L3 and L4, can also contribute to linezolid resistance. These modifications alter the ribosome’s structure in a way that reduces linezolid binding without significantly impairing ribosomal function [79]. Furthermore, a mutation in the *rrl5* gene, which encodes the 23S rRNA in the ribosome’s 50S subunit, alters the linezolid target site within the V domain of the 23S rRNA, thereby reducing the drug’s efficacy. In *S. aureus* strains, the T2500A mutation in the *rrl5* gene results in resistance, with MIC values ranging from 8 mg/L to 16 mg/L, exceeding the CLSI breakpoint MIC value of ≥8 mg/L [80]. These modifications effectively alter ribosomal binding sites, rendering antibiotics ineffective.

#### 3.1.7. Efflux Pumps

The *S. aureus* genome contains over 30 potential efflux pumps [81]. The removal of tetracycline in *S. aureus* is facilitated by membrane proteins such as Tet(K), Tet(L), Tet(38), Tet(42), Tet(43), Tet(45), and Tet(63), which are powered by a proton pump and belong to the major facilitator superfamily (MFS). The Tet(K) protein, featuring 14 transmembrane segments, confers resistance to several tetracyclines, including tigecycline, doxycycline, and eravacycline, but not to minocycline [63]. Tet(K) demonstrates resistance to tetracycline (MICs ≤ 32–128 mg/L) and doxycycline (MICs ≤ 2–4 mg/L) [82].

MgrA (multiple gene regulator A) regulates four multidrug resistance efflux pumps in *S. aureus* belonging to the MFS family: NorA, NorB, NorC, and Tet(38). MgrA serves as a key regulatory protein that modulates the expression of various genes, including those encoding efflux pumps [83]. NorA, extensively studied, is known for conferring resistance to fluoroquinolones [84]. NorB and Tet(38) provide resistance to both fluoroquinolones and tetracyclines [85], while NorC also confers resistance to fluoroquinolones [83]. The Nor family of pumps expels norfloxacin, ciprofloxacin, moxifloxacin, and sparfloxacin [86].

Methicillin-resistant *Staphylococcus aureus* (MRSA) strains frequently harbor plasmid-borne genes that confer resistance to various antiseptics and disinfectants, such as quaternary ammonium compounds (QACs) and chlorhexidine, as well as to DNA intercalating agents like acriflavine and ethidium bromide. The efflux pumps QacA/B and NorA play a crucial role in removing these compounds from the bacterial cell. QacA is located on the plasmid pSK1, while QacB is found on the plasmid pSK23. Both pumps use the proton motive force to transport drugs out of the cell. The expression of these efflux pump genes is regulated by the protein QacR [87,88].

Other efflux pumps classified under the MFS include SdrM, MdeA, and LmrS, which are located on the bacterial chromosome [81]. LmrS, a multidrug transporter, effectively removes a variety of antibiotics, including chloramphenicol, lincomycin, streptomycin, kanamycin, linezolid, and fusidic acid [89]. The MdeA efflux pump, powered by the proton motive force, expels several drugs, including ciprofloxacin, macrolides, fusidic acid, and anthracycline drugs such as doxorubicin and daunorubicin, contributing to multidrug resistance [90,91]. The SdrM efflux pump removes norfloxacin, acriflavin, ethidium bromide, and biocides from bacterial cells. SdrM shares structural similarities with other efflux pumps, such as NorB and QacA [92]. A tetracycline-specific efflux protein coded by *S. aureus* strain SA01, which shares 73.0% similarity with Tet(K), has been identified in chickens [93].

Efflux pumps in *S. aureus* belong to the small multidrug resistance (SMR) family, including QacC, also known as Ebr, QacD, or Smr. The gene encoding this efflux pump is located on plasmids and facilitates the removal of quaternary ammonium compounds, disinfectants, and ethidium bromide [47]. Another SMR efflux pump, SepA, contains four transmembrane segments and provides low-level resistance to various antiseptics and dyes [94].

The MepA efflux pump, a member of the multidrug and toxin extrusion (MATE) family, is encoded by the chromosomal *mepA* gene and is composed of 451 amino acids. It was the first multidrug transporter of the MATE family identified in *S. aureus* [95]. The expression of *mepA* is regulated by the *mepRAB* gene cluster, including MepR, a MarR family repressor that controls MepA efflux activity [96].

**Figure 5:**
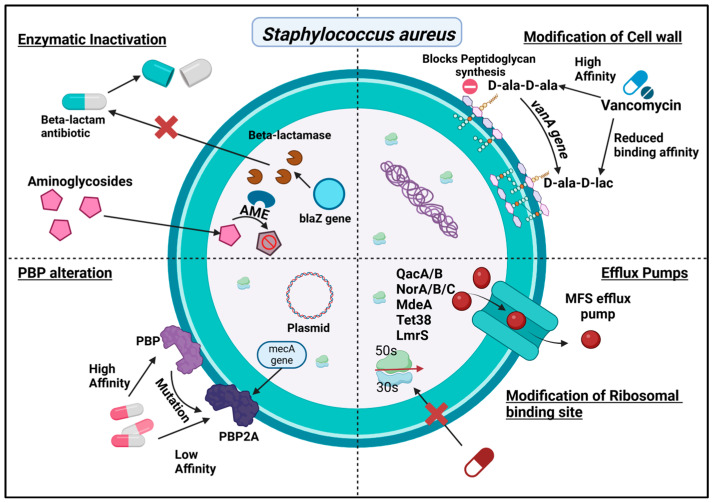
Mechanisms of Antibiotic Resistance in *Staphylococcus aureus*.

SAV1866, an efflux pump in the ATP-binding cassette (ABC) family, plays a significant role in drug resistance. The 3.0 Å crystal structure of SAV1866, a symmetrical homodimer, serves as a homology model for studying ABC proteins in humans [97]. Another ABC pump, AbcA, provides resistance to hydrophobic β-lactams and is involved in cell wall autolysis. Its expression is regulated by the MgrA regulator. Overexpression of AbcA in *S. aureus* strain MW2 caused a 12.5-fold decrease in MIC for teicoplanin and a 3.1-fold decrease in MIC for telavancin, indicating its potential role in resistance to these antibiotics [98]. Figure 5 summarizes the major mechanisms employed by *Staphylococcus aureus* to resist the effects of antibiotics, divided into four categories: enzymatic inactivation, modification of the cell wall and ribosomal binding site, efflux pumps, and PBP alteration.

### 3.2. Streptococcus pneumoniae

*Streptococcus pneumoniae*, or pneumococcus, is a Gram-positive bacterial species belonging to the Streptococcaceae family. It is responsible for various infections, including pneumonia, meningitis, and otitis media. The bacterium colonizes the mucosal lining of the respiratory tract, leading to both invasive and non-invasive illnesses. Vulnerable groups, such as children, the elderly, and individuals with compromised immune systems, are particularly susceptible to its effects [99]. Pneumococci are transmitted from person to person via respiratory droplets, and epidemics can occur in closed populations [100]. The bacterium has successfully adapted to the challenges posed by various antibiotic classes and is now showing signs of resistance to the immune effects of extensive antibiotic use [99].

Treatment for *S. pneumoniae* infections typically involves antibiotics, with penicillin as the first-line treatment for susceptible strains. However, the rise in antibiotic resistance, particularly to β-lactam antibiotics such as penicillins, cephalosporins, and carbapenems, as well as other drug classes like macrolides, fluoroquinolones, and sulfamethoxazole-trimethoprim, has made treatment more complex. In severe cases, such as pneumococcal meningitis, vancomycin—an antibiotic that inhibits cell wall synthesis—is added to the standard treatment regimen [101].

Two primary pneumococcal vaccines are available: the polyvalent pneumococcal polysaccharide vaccine (PPV) and the pneumococcal conjugate vaccine (PCV) [102]. Following the introduction of PCV7 in the United States, the incidence of invasive pneumococcal disease caused by penicillin-resistant and multidrug-resistant *S. pneumoniae* strains declined. For example, between 1999 and 2004, the rate of invasive disease caused by penicillin-resistant strains decreased from 6.3 cases per 100,000 to 2.7 cases per 100,000 [103]. Despite a century of extensive research and the development of effective treatments, pneumococcus-related respiratory tract infections remain a significant global concern [104]. The World Health Organization (WHO) reports that pneumococcal disease causes approximately 1.6 million deaths annually across all age groups worldwide.

**Figure 6:**
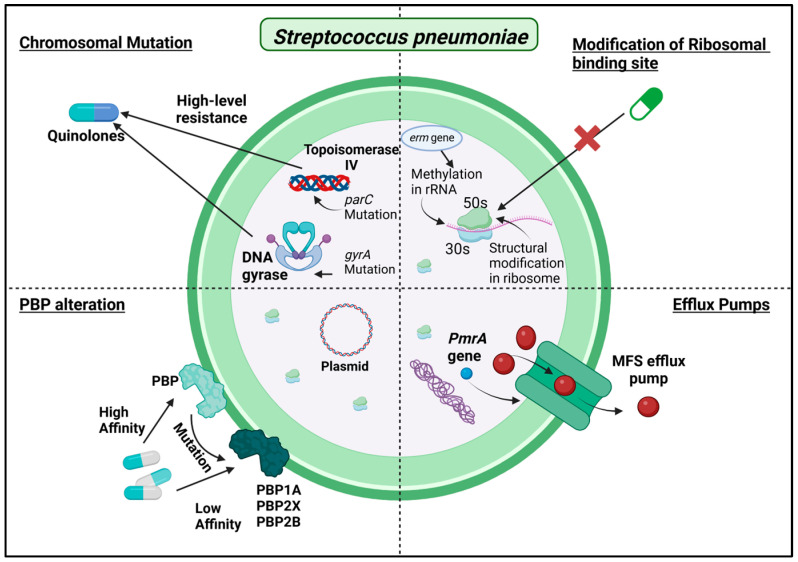
Mechanisms of Antibiotic Resistance in *Streptococcus pneumoniae*.

As naturally transformable organisms, no strains of *S. pneumoniae*-producing beta-lactamase have been identified [105]. The generation of aminoglycoside-modifying enzymes is not typically associated with *S. pneumoniae* and is relatively uncommon, particularly compared to other bacteria like *Staphylococcus aureus* or Enterococcus species. Figure 6 summarizes the major mechanisms employed by *Streptococcus pneumoniae* to resist the effects of antibiotics.

#### 3.2.1. Target Modification

(a) PBP alteration

*Streptococcus pneumoniae* has the natural ability to undergo genetic transformation, enabling it to take up and incorporate DNA from its environment into its genome. This process can lead to the development of mosaic structures in its penicillin-binding protein (PBP) genes. These mosaic PBPs result from interspecies homologous recombination, where DNA sequences from different bacterial species are integrated into the pneumococcal genome [106,107]. *S. pneumoniae* possesses five high-molecular-weight PBPs: PBP1A, PBP1B, PBP2A, PBP2B, and PBP2X [108].

The modified PBPs in pneumococci exhibit significantly reduced affinity for nearly all β-lactam antibiotics, including third-generation cephalosporins [107]. PBPs 1A, 2X, and 2B are particularly associated with high levels of penicillin resistance [109]. *S. pneumoniae* develops resistance to β-lactam antibiotics by acquiring mutations that alter the stability and charge of the active site in these crucial enzymes. For example, the *S. pneumoniae* 5259 strain shows resistance due to changes in polarity and charge distribution at the entrance of the catalytic gorge caused by a mutation from Gln552 to Glu in pneumococcal PBP2X [110]. Structural analyses of PBP1A sequences from drug-resistant clinical strains from various countries revealed a similar resistance pattern characterized by the presence of mutational hotspots that alter the polarity and accessibility of the PBP1A active site [111].

An interspecies transformation experiment revealed that PBP2X and PBP1A are essential for developing cefotaxime resistance. PBP2X plays the primary role, while PBP1A supports it as a secondary factor. Additionally, the experiment demonstrated that PBP2B is the main protein responsible for piperacillin resistance. Introducing PBP1A into the R6 transformant strain increased the MIC for cefotaxime and also induced resistance to piperacillin and oxacillin. Thus, PBP1A acts as a secondary resistance determinant, enhancing resistance to cefotaxime as well as other penicillin-class antibiotics [112].

(b) Chromosomal mutation

Quinolones inhibit bacterial DNA gyrase and topoisomerase IV [113]. DNA gyrase is essential for DNA replication, specifically the separation of DNA strands, while topoisomerase IV is necessary for the partitioning of replicated chromosomal DNA, enabling it to be packaged within the cell [114]. In *S. pneumoniae*, quinolone resistance develops through two stepwise chromosomal mutations. The first-step mutation occurs in the *parC* gene of topoisomerase IV, leading to low-level quinolone resistance. The second-step mutation occurs in the *gyrA* gene of DNA gyrase, resulting in high-level resistance [115].

Fluoroquinolones, such as ciprofloxacin and ofloxacin, exhibit limited effectiveness against *S. pneumoniae* because their MICs are at or exceed the breakpoint. Resistance to sparfloxacin in *S. pneumoniae* developed following two mutations: one in the *gyrA* gene and another unidentified mutation, resulting in an MIC of 4 mg/mL [116].

(c) Modification of ribosomal binding site

*Streptococcus pneumoniae* exhibits resistance to macrolide–lincosamide–streptogramin (MLS) antibiotics. The MLS resistance mechanism involves the *erm* gene, which encodes an S-adenosylmethionine-dependent methylase. This enzyme methylates adenine residues within the peptidyl transferase domain of the 23S rRNA, inducing structural alterations in the ribosome. These changes reduce the binding affinity of MLS antibiotics to the rRNA [117]. Penicillin-resistant *S. pneumoniae* shows resistance to erythromycin, azithromycin, clarithromycin, and clindamycin, with MICs exceeding 64 µg/mL [118].

Tetracycline resistance in *S. pneumoniae* is mediated by ribosomal protection through the *tet*(M) and *tet*(O) genes. Tetracyclines act by binding to the A-site or P-site of the bacterial ribosome’s 30S subunit, preventing the binding of aminoacyl-tRNA to the A-site and thus hindering protein synthesis [119].

#### 3.2.2. Efflux Pumps

*Streptococcus pneumoniae* employs efflux mechanisms that contribute to fluoroquinolone resistance. The *pmrA* gene, a member of the major facilitator superfamily of efflux pumps, plays a role in multidrug efflux resistance in pneumococci by regulating the expression of these pumps [120]. This mechanism reduces susceptibility to certain fluoroquinolones with breakpoint MICs of ≥16 μg/mL, such as norfloxacin (MIC, 32 μg/mL) and ciprofloxacin (MIC, 64 μg/mL) [121].

Additionally, the *mefE* gene encodes macrolide efflux pumps, which actively expel macrolides from the bacterial cell. This efflux mechanism confers resistance to various macrolides in *S. pneumoniae*, including erythromycin (MIC, 64 μg/mL), clarithromycin (MIC, 32 μg/mL), and azithromycin (MIC, 96 μg/mL) [122].

#### 3.2.3. Biofilm Formation

Numerous genes with varied functions play a role in the formation and dispersal of *S. pneumoniae* biofilms. These biofilms create an optimal setting for horizontal gene transfer (HGT) in *S. pneumoniae* [123].

### 3.3. Enterococcus faecium

Enterococci, Gram-positive cocci, typically exist as commensal organisms within the gastrointestinal tracts of humans and animals. *Enterococcus faecium*, a notable member of the *Enterococcaceae* family, has transitioned from being regarded as a harmless component of the gut flora to a significant cause of hospital-acquired infections [124]. *E. faecium* is responsible for various infections, including urinary tract infections (UTIs), intra-abdominal infections, and endocarditis. In Europe, enterococci are identified as the second leading cause of wound infections and UTIs and rank third in causing bacteremia [125,126]. In the United States, approximately 12% of hospital-acquired infections are attributed to *Enterococcus* species [127]. These infections are particularly concerning in healthcare settings due to the bacterium’s ability to resist antibiotics [128].

*E. faecium* possesses numerous transposons and plasmids that confer resistance to a broad range of antibiotics, including erythromycin, gentamicin, kanamycin, streptomycin, tetracycline, and vancomycin [124]. A majority of *E. faecium* strains show resistance to vancomycin, a glycopeptide antimicrobial. Among the six phenotypes of *E. faecium* resistance, the VanA and VanB types are the most frequently reported [129]. Furthermore, enterococci are considered probable reservoirs of drug-resistance genes and may facilitate the dissemination of these genetic determinants to other Gram-positive pathogens, exacerbating the challenge of antimicrobial resistance in healthcare facilities [130].

The presence of enterococci in pasteurized cheeses, fermented dairy products, beef, poultry, pork, and other meat products has been highlighted by Giraffa Giorgio. When these bacteria contaminate food products, resistant strains can spread to humans via the food chain, leading to colonization [131]. A prospective laboratory-based study monitoring resistant *Enterococcus* isolates from both patients and cheese samples identified strains with significant resistance to kanamycin and gentamicin in both French raw milk cheeses and hospitalized individuals. This finding suggests that cheeses might act as a reservoir for antibiotic-resistant *Enterococcus*, possessing traits that enable their persistence and dissemination within the community [132].

Considering the role of enterococci in the gut microbiota, their entry into the food chain, their contribution to antibiotic resistance and the spread of resistance genes, and their association with foodborne diseases, these bacteria have become significant hospital-acquired pathogens. They particularly affect immunocompromised patients and those in intensive care units [131].

**Figure 7:**
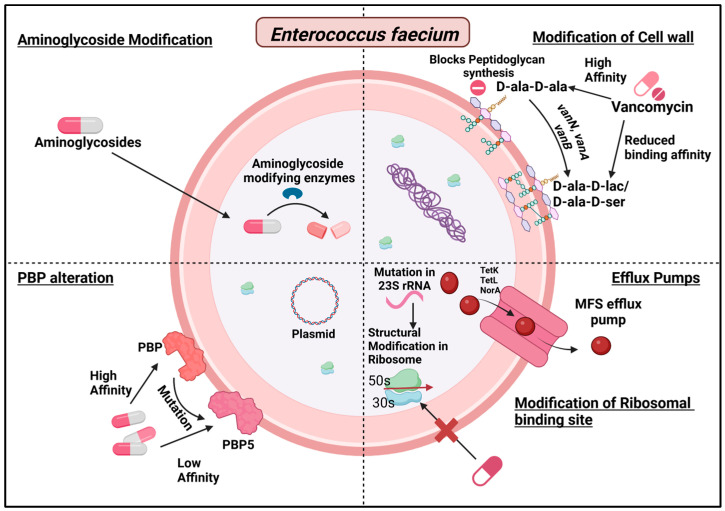
Mechanisms of antibiotic resistance in *Enterococcus faecium*.

The following section will explain the resistance mechanisms produced by enetrococci, and Figure 7 summarizes various strategies employed by *E. faecium* to evade the effects of antibiotics.

#### 3.3.1. Aminoglycoside Modifying Enzymes

*Enterococcus faecium* exhibits emerging resistance genes, such as *aph(2″)-Ic* and *aph(2″)-Id*, in vancomycin-resistant strains, along with the *aph(2″)-Ib* gene, which is responsible for amikacin resistance [133]. Approximately half to three-fifths of recent clinical *E. faecium* isolates exhibit significant resistance to streptomycin, while resistance to gentamicin varies between 20% and 80% across different countries [134].

*E. faecium* shows intrinsic resistance to tobramycin and kanamycin, with MICs reaching up to 1000 µg/mL due to chromosomal enzymes AAC(6′)-Ii and EfmM. Therefore, gentamicin and streptomycin are preferred for treating severe *E. faecium* infections. High-level streptomycin resistance arises from mutations in the S12 ribosomal protein or the acquisition of genes encoding the ANT(3″)-Ia or ANT(6′)-Ia enzymes [134,135]. Additionally, *E. faecium* strains harboring the *aph(2″)-Ie* gene exhibit significant resistance to gentamicin and streptomycin, with MICs exceeding 1000 µg/mL. Another strain, E503, demonstrated strong resistance to netilmicin. However, no high-level resistance was detected for kanamycin, dibekacin, or tobramycin [136].

#### 3.3.2. Modification of Cell Wall

*Enterococcus faecium* develops resistance to the glycopeptide antibiotics vancomycin and teicoplanin by altering the pentapeptide precursors in its peptidoglycan. This alteration involves replacing d-alanine with d-lactate or d-serine, which significantly reduces the antibiotics’ binding affinity [137]. The vanN operon is solely responsible for producing d-Ala-d-Ser-ending precursors in *E. faecium* [138].

VanA strains exhibit high-level resistance to both teicoplanin and vancomycin, with MIC values exceeding 64 mg/L. In contrast, VanB strains display a range of vancomycin resistance levels while remaining susceptible to teicoplanin. The expression of the operon’s genes is regulated by the vanRS two-component system. Precursors ending in d-Ala-d-Lac exhibit a 1000-fold reduction in binding affinity compared to d-Ala-d-Ala, resulting in high-level vancomycin resistance (MIC > 16 mg/L). Conversely, precursors with d-Ala-d-Ser show a seven-fold reduction in vancomycin binding affinity, leading to moderate resistance with MIC values ranging from 8 to 16 mg/L [134,139].

#### 3.3.3. Biofilm Formation

Biofilm formation is a key virulence factor of *Enterococcus*. The *esp* gene significantly contributes to this process, making it a critical element in the pathogenesis of infections [140]. A recent investigation of *Enterococcus faecium* strains from a pig farm environment revealed resistance to ampicillin, vancomycin, linezolid, and high doses of gentamicin. The analysis demonstrated that vancomycin-resistant *E. faecium* strains exhibited an enhanced ability to form biofilms, which aids their survival under elevated environmental stress. This increased resistance facilitates the spread of *E. faecium* infections among farmers, veterinarians, and workers in breeding farms and slaughterhouses. Furthermore, the biofilm matrix impedes antibiotic penetration, thereby enhancing antibiotic tolerance [141].

#### 3.3.4. Modification of Ribosomal Binding Site

*Enterococcus faecium* develops resistance by altering ribosomal RNA or related proteins, which hinders antibiotics’ ability to bind and inhibit bacterial protein synthesis. Resistance to linezolid (MIC90: 2–4 mg/L) arises through mutations in the domain V region of the 23S rRNA gene, particularly the G2576T mutation, which decreases the binding affinity of linezolid to the rRNA, reducing its effectiveness. Additionally, the acquisition of the chloramphenicol-florfenicol resistance (cfr) gene, which encodes a methyltransferase that methylates adenine at position 2503 of the 23S rRNA, contributes to resistance. This modification not only confers resistance to linezolid but also to other antibiotic classes, including phenicols, lincosamides, and pleuromutilins.

Notably, high-level resistance to fluoroquinolones is becoming increasingly common in hospital-adapted *E. faecium* clinical isolates. This resistance is attributed to point mutations in the *gyrA* and *parC* genes, which encode the A subunits of DNA gyrase and topoisomerase IV, respectively [134].

#### 3.3.5. PBP Alteration

Sometimes, bacteria increase the production of penicillin-binding proteins (PBPs), reducing the effectiveness of β-lactam antibiotics. With more PBPs present, not all of them can be blocked by the antibiotic, allowing the bacteria to continue synthesizing their cell walls. Studies have revealed that resistance to β-lactams, primarily ampicillin, in *Enterococcus faecium* operates through the same mechanism. This resistance is attributed to PBP5 overexpression and mutations that reduce its affinity for β-lactams [142]. While some strains rely primarily on PBP5 overexpression for resistance, others employ both mechanisms—overexpression and reduced antibiotic affinity—to achieve extreme levels of resistance [143].

#### 3.3.6. Efflux Pumps

A limited number of drug efflux pumps, including MsrC, Tet(K), and Tet(L), have been identified in *E. faecium*. The *tet*(K) and *tet*(L) genes encode efflux pumps that expel tetracyclines from the cell, with the exception of tigecycline. These pumps belong to the major facilitator superfamily (MFS) and operate by utilizing the proton motive force to transport tetracyclines out of the bacterial cell [134]. Additionally, evidence suggests that *E. faecium* possesses an efflux pump similar to NorA, which may contribute to its resistance to the hydrophilic fluoroquinolones sparfloxacin and norfloxacin [144].

Table 1 summarises the key resistance mechanisms in key Gram-positive bacteria, the diseases they are associated with, the effective antibiotics used against them and key studies reporting these findings.

## 4. Current Treatment Options

In the last decade, several alternative antibiotics have been developed to address resistance in existing treatments, particularly for priority pathogens identified by the WHO. Recently developed cephalosporins include ceftobiprole, ceftaroline, cefiderocol, and ceftolozane (paired with tazobactam). Ceftobiprole, a fifth-generation cephalosporin, is the first β-lactam effective against methicillin-resistant *Staphylococcus aureus* (MRSA) and vancomycin-resistant *Staphylococcus aureus* (VRSA). It achieves this by efficiently binding to altered PBPs such as PBP 2a and PBP 2x, which are critical in resistant bacteria like *Streptococcus pneumoniae* [145].

Delafloxacin, approved in 2017, is a fluoroquinolone with broad-spectrum activity against resistant Gram-positive and Gram-negative bacteria, including MRSA and *Pseudomonas aeruginosa*. Its availability in both oral and intravenous forms makes it unique among antibiotics used to treat acute bacterial skin and skin structure infections (ABSSSIs) [146]. Another novel drug, lefamulin, a pleuromutilin antibiotic, was approved by the FDA in 2019 and the European Commission in 2020 for treating community-acquired bacterial pneumonia (CABP). Lefamulin is effective against Gram-positive pathogens such as *Streptococcus pneumoniae* and *Staphylococcus aureus*. It is the first pleuromutilin derivative approved for both oral (PO) and intravenous (IV) use in humans. Pleuromutilins, including their derivatives, inhibit bacterial protein synthesis by binding uniquely to the peptidyl transferase center of the 50S ribosomal subunit. Their tricyclic core and C14 side chains disrupt correct tRNA positioning through an “induced-fit mechanism”, differing from other antibiotics targeting protein synthesis [147].

These newer antibiotics are often designed to bypass existing resistance mechanisms, providing better outcomes in resistant infections. However, their use is typically reserved as a last-line treatment to prevent the rapid emergence of resistance. The effectiveness of recently developed antibiotics is closely tied to their usage patterns and the potential for resistance development. The limited number of new antibiotics targeting priority pathogens in the past decade—only one or two in clinical development—underscores a significant unmet clinical need. This scarcity makes it challenging to determine whether these antibiotics are inherently more effective or simply appear so because widespread resistance has not yet emerged. While limited use may delay resistance development, it also highlights the urgent need for continued innovation and diversification in antibiotic research and development to address persistent gaps in treatment options.

## 5. Future Perspectives

Antimicrobial resistance (AMR) continues to be a serious global health concern, particularly with Gram-positive bacteria such as *Staphylococcus aureus*, *Streptococcus pneumoniae*, and *Enterococcus faecium*. These pathogens are highlighted in the 2024 World Health Organization (WHO) high-priority list for their ability to cause severe infections and their growing resistance to standard antibiotics [14].

The extensive application of antibiotics in medical treatment has led to drug resistance and heightened the risk of the emergence of super-resistant bacteria [148]. Antimicrobial resistance in bacteria through horizontal gene transfer (HGT) is an ongoing process driven by genetic mutations and the spread of drug resistance genes between bacteria. Human activities significantly contribute to this problem, impacting people globally. To address this urgent issue, coordinated international efforts are essential. Adopting thorough strategies is crucial to effectively combat bacterial resistance [137].

Developing new antibiotics targeting the pathogens listed in the WHO Bacterial Priority Pathogens List (BPPL) presents significant scientific and commercial challenges. Although 42 antibiotics are under clinical development, only 11 of these have the potential to treat pathogens on the WHO’s critical threat list. A significant scientific challenge in antibiotic development is the complexity of discovering molecules that are selectively harmful to bacteria without causing damage to human cells. These antibiotics must not only efficiently inhibit bacterial growth or kill bacteria but also avoid mechanisms that promote resistance. Antibiotics are prescribed for short durations and priced lower than chronic disease treatments, making them less profitable. New antibiotics are reserved as a last resort to prevent resistance, limiting sales and market growth even after regulatory approval, discouraging private investment, and creating funding gaps in R&D [149].

The development and availability of new antibiotics have not kept up with the rapid progression of antimicrobial resistance [137]. Identifying antibiotic-resistant genes (ARGs) presents significant challenges due to the need for precise accuracy in practical treatments and the robustness to rapidly identify issues. Current laboratory characterization and diagnostic methods often fall short, yielding inconsistent results influenced by varying environmental and laboratory conditions. Consequently, the use of artificial intelligence (AI) techniques, particularly machine learning (ML) and deep learning (DL), has become essential [150].

Applying emerging therapies for early identification of infectious diseases and distinguishing between infectious and non-infectious conditions is critical in addressing antibiotic resistance. In 2019, Yelin et al. developed a model for detecting urinary tract infections (UTIs) that leveraged personal clinical histories through a machine learning-based AMR prediction approach. They analyzed ten years of data from 0.7 million community-acquired UTI cases, uncovering a strong link between antimicrobial resistance and factors such as demographic details, previous urine culture results, and patients’ past antibiotic usage [151]. It is crucial to monitor environmental media such as wastewater, agricultural waste, food, and water to detect new antibiotic resistance genes (ARGs), identify gene exchange hotspots, and understand ARG pathways and human exposure risks. Traditional methods often result in many false negatives, as they rely on matching sequences to existing database entries. Deep learning offers a solution to this problem. In a study, DeepARG-SS and DeepARG-LS models were developed to handle short-read and full-length gene sequences, respectively. These models demonstrated high accuracy in predicting ARGs and consistently produced fewer false negatives [152]. Machine learning methods are also applied in antimicrobial resistance (AMR) to detect antibiotic properties in drugs aimed at humans, which may help prevent the development of resistant bacteria. A machine-learning model was trained to categorize drug compounds using heterologous training sets, incorporating both peptide and non-peptide antimicrobial compounds to expand the training dataset. This approach facilitated the identification of antimicrobial activity in drugs intended for human use [153]. AI techniques like ML and DL offer powerful tools for addressing the challenges of antimicrobial resistance by improving diagnostic accuracy, predicting resistance patterns, and identifying new antimicrobial agents.

Additionally, the role of genomics in AMR should be explored further. Genomics is vital for understanding how antimicrobial resistance (AMR) works, as it provides in-depth insights into the genetic basis of resistance mechanisms. The interactions between humans, pets, livestock, and wildlife differ significantly in urban, rural, and remote settings. Current surveillance of antimicrobial-resistant bacteria from animals or food primarily relies on microbiological and phenotypic methods, with genomic techniques mainly used to explore atypical AMR patterns [154]. Genomics isn’t as widely used for detecting and monitoring bacterial AMR compared to other applications like strain typing and phylogenetic analysis. This delay is due to phenotypic testing being quicker than genotypic testing and the lack of global standards for genomic detection, making it hard to compare results across labs. However, whole-genome sequencing (WGS) has become crucial in AMR surveillance. It helps researchers pinpoint where resistant bacteria originate and how they spread in healthcare settings and the environment. Sherry and colleagues developed and confirmed a computational system to identify antimicrobial resistance (AMR) traits in various bacteria. AMRFinderPlus (https://github.com/ncbi/amr (accessed on 24 October 2024)), an ISO-certified genomic software solution, employs diverse search techniques to accurately detect AMR genes and mutations [155]. As genetic information becomes more available and incorporated into healthcare networks, it will significantly contribute to addressing the worldwide issue of antimicrobial resistance (AMR) [156].

While emerging technologies and novel antibiotic development are pivotal in combating antimicrobial resistance (AMR), addressing this global health crisis requires coordinated international collaboration. Collaborative efforts among nations, scientific communities, and stakeholders are essential to ensure equitable access to resources, harmonized policies, and shared expertise. In 2015, FAO, OIE, and WHO launched the Global Action Plan (GAP) on Antimicrobial Resistance, urging countries to adopt multisectoral national action plans (NAPs) aligned with GAP principles [157]. Ahead of the 2024 UN General Assembly High-Level Meeting on AMR, the AMR Industry Alliance urges the UN and its Member States to intensify efforts against AMR. They emphasize collaboration with public and private stakeholders to prioritize and implement risk-based solutions to significantly reduce its spread. Despite ongoing investments, funding for AMR research is declining, especially in critical development stages, leading to a weak antibiotic pipeline. To address this, the AMR Industry Alliance invests USD 2 billion annually, alongside the USD 1 billion AMR Action Fund by the private sector, to support new antimicrobial R&D [158]. The AMR Industry Alliance’s Stewardship Prize initiative highlights successful, innovative antimicrobial stewardship strategies in low- and middle-income countries (LMICs), aiming to inspire similar efforts globally. In 2016, the WHO and the United Nations General Assembly endorsed the global and institutional implementation of antimicrobial stewardship programs (AMS) [159]. Other collaborative efforts, like the Global Antimicrobial Resistance and Use Surveillance System (GLASS), focus on integrating data across sectors to understand AMR and AMC (antimicrobial consumption) and combat AMR [160]. Groups such as the Antibacterial Resistance Leadership Group (ARLG) prioritize research on Gram-positive pathogens like MRSA and VRE (vancomycin-resistant *Enterococcus*) to improve treatment strategies and diagnostics [161].

## 6. Conclusions

In conclusion, the WHO’s 2024 priority pathogen list underscores the urgent need for focused research on high-priority bacteria. Gram-positive infections have historically been easier to treat due to their less protective membrane barriers, which make them more susceptible to antibiotics. However, antimicrobial resistance (AMR) in these bacteria is evolving, with species such as *Staphylococcus aureus*, *Streptococcus pneumoniae*, and *Enterococcus faecium* employing diverse resistance strategies. This variety in resistance mechanisms necessitates targeted approaches to combat AMR effectively.

While Gram-positive infections are generally easier to treat than Gram-negative ones, rising resistance has significantly reduced the efficacy of many antibiotics. Drugs that were once commonly used are now less effective or less frequently prescribed. Addressing this issue requires a concerted effort involving antimicrobial stewardship and innovative research. The integration of artificial intelligence (AI), machine learning, deep learning, and genomics offers promising tools to preserve antibiotic effectiveness and develop novel solutions.

## Figures and Tables

**Table 1:** Overview of Bacterial Species, Associated Diseases, Effective Antibiotics, and Resistance Mechanisms.

Bacterial Species	Diseases Caused by These Bacteria	Antibiotics	Bacterial Resistance Mechanism	Reference
*Staphylococcus aureus*	Skin infections, soft tissue infections, pneumonia, sepsis	Beta-lactams-cloxacillin, ceftriaxone, amoxicillin	Inactivation by beta-lactamases	[58,59]
		Aminoglycosides- Lividomycin, Amikacin, Neomycin, Kanamycin, Streptomycin, Spectinomycin, Gentamicin, tobramycin	Aminoglycoside Modification by aminoglycoside modification enzymes.	[60,61,62,63]
		Vancomycin, Gentamicin, Daptomycin	Alteration of target by modification of cell wall	[66,67,68,71,72]
		Penicillin-G, Oxacillin, Ampicillin, Piperacillin, Cefaclor, Cefotaxime, Cephalexin, Cefoxitin	Penicillin-binding protein alteration	[74,75,76]
		Tetracyclines, Minocycline, Erythromycin, Quinpristin, Dalfopristin, Linezolid	Modification of Ribosomal binding sites	[63,77,78,79,80]
		Tigecycline, Doxycycline, Eravacycline, fluoroquinolones, Norfloxacin, Ciprofloxacin, Moxifloxacin, Sparfloxacin, Chloramphenicol, Lincomycin, Streptomycin, Kanamycin, Linezolid, Macrolides, Doxorubicin, Daunorubicin, Acriflavin	Efflux Pumps	[82,84,86,89,90,91,92]
*Streptococcus pneumoniae*	Pneumonia, Meningitis, otitis media	Piperacillin, Cefotaxime, Oxacillin, and all other beta-lactam antibiotics	Penicillin-Binding Protein alteration	[99,107,112]
		Quinolones, Ciprofloxacin, Ofloxacin	Chromosomal Mutation	[115,116]
		Macrolide, Lincosamide, Streptogramins, Erythromycin, Azithromycin, Clarithromycin, Clindamycin	Modification of Ribosomal binding site	[117,118]
		Norfloxacin, Ciprofloxacin, Macrolide, Erythromycin, Clarithromycin, Azithromycin	Efflux Pumps	[122]
*Enterococcus faecium*	Urinary tract infections, Intra-abdominal infections, Wound infections, Endocarditis	Amikacin, Streptomycin, Gentamicin, Tobramycin, Kanamycin	Aminoglycoside Modification by aminoglycoside modification enzymes.	[125,126,134,135,136]
		Vancomycin, Teicoplanin	Modification of cell wall	[134,137,139]
		Ampicillin, Vancomycin, Linezolid, Gentamicin	Biofilm formation	[134]
		Linezolid, Phenicols, Lincosamide, Pleuromutilins	Modification of ribosomal binding sites	[134]
		Beta-lactams	Penicillin-binding protein	[143]
		Tetracyclines (Except Tigecycline), Sparfloxacin, Norfloxacin	Efflux Pumps	[134,144]

## Data Availability

Not applicable.
